# LTP-1, a novel antimitotic agent and Stat3 inhibitor, inhibits human pancreatic carcinomas *in vitro* and *in vivo*

**DOI:** 10.1038/srep27794

**Published:** 2016-06-09

**Authors:** Han-Li Huang, Min-Wu Chao, Chung-Chun Chen, Chun-Chun Cheng, Mei-Chuan Chen, Chao-Feng Lin, Jing-Ping Liou, Che-Ming Teng, Shiow-Lin Pan

**Affiliations:** 1 The Ph.D. Program for Cancer Biology and Drug Discovery, College of Medical Science and Technology, Taipei Medical University, Taipei, Taiwan; 2 Pharmacological Institute, College of Medicine, National Taiwan University, Taipei, Taiwan; 3 Ph.D. Program for the Clinical Drug Discovery from Botanical Herbs, College of Pharmacy, Taipei Medical University, Taipei, Taiwan; 4 Graduate Institute of Pharmacognosy, College of Pharmacy, Taipei Medical University, Taipei, Taiwan; 5 Department of Internal Medicine, Division of Cardiology, Shuang Ho Hospital, Taipei Medical University, New Taipei City, Taiwan; 6 School of Pharmacy, College of Pharmacy, Taipei Medical University, Taipei, Taiwan; 7 Department of Pharmacology, College of Medicine, Taipei Medical University, Taipei, Taiwan

## Abstract

Pancreatic cancer is the leading cause of cancer death worldwide with a poor survival rate. The objective of this study was to determine the mechanism of action of a novel antimitotic and Stat3 inhibitor, LTP-1, on human pancreatic cancer *in vitro* and *in vivo*. We found that LTP-1 inhibited pancreatic cancer cell growth and viability with significant G_2_/M arrest and disruption of microtubule dynamics. LTP-1 also caused G_2_/M arrest-independent Stat3 dephosphorylation along with ERK activation, which indicated the possible dual function of LTP-1. Long-term treatment of LTP-1 also induced polyploidy, activated caspases, induced subG_1_ cell population, and therefore, triggered pancreatic cancer cell apoptosis. Finally, we used an *in viv*o xenograft model to demonstrate that LTP-1 suppressed the growth of pancreatic adenocarcinoma. In summary, our data suggest that LTP-1 may alter microtubule dynamics, which ultimately causes polyploidy and apoptosis, thereby inhibiting pancreatic cancer growth *in vitro* and *in vivo*. This study provides evidence that LTP-1 could be a potential therapeutic agent for further development of pancreatic cancer treatment.

Pancreatic cancer is the estimated fourth leading cause of cancer death in the United States according to a report of the American Cancer Society^1^. The current 5-year relative survival rate for pancreatic cancer is 7%; however, in more than half of the cases, the cancer is diagnosed at a later stage, for which the 5-year survival is only 2%^1^. Although many therapeutic agents (such as gemcitabine and nab-paclitaxel) have been developed for pancreatic cancer treatment, the survival rate has not improved to a desirable extent^2^. Therefore, developing anticancer agents for treating pancreatic cancer is an essential and urgent medical need. Recent evidence has shown that signal transducer and activator of transcription 3 (Stat3) is a potent therapeutic target against pancreatic cancer^3^.

Microtubules, composed of α/β-tubulin heterodimers, are key components of the mitotic spindle and essential for chromosome segregation during mitosis and cell division^4^. Abundant evidence indicates that microtubule-targeting agents are effective antitumor agents since they interfere with the dynamics of mitotic spindles, leading to cell cycle arrest and death of cancer cells^5^. Microtubule-targeting agents are classified into two major groups: microtubule-destabilizing agents (e.g. vinca alkaloid) and microtubule-stabilizing agents (e.g. paclitaxel). These agents bind to tubulin and affect G_2_/M transition and subsequently induce G_2_/M arrest and apoptosis, which are controlled by coordination of cyclins, cyclin-dependent kinases (CDKs) and CDK inhibitors^6^. During the G_2_/M transition, cdc2 is dephosphorylated on tyrosine-15 residue by cdc25C and phosphorylated by the cyclin activating kinase (CAK) on threonin-161 residue to activate the M-phase-promoting factor, cdc2/cyclin B1 complex, which controls mitotic progression^7^. Microtubule binding agents are also able to act synergistically with gemcitabine to prolong the survival of pancreatic cancer patients^8^,^9^.

In recent times, growing evidence has suggested the potential involvement of Stat3 in malignant transformation, angiogenesis, tumor growth, and metastasis of human pancreatic cancer, indicating that Stat3 may be targeted for pancreatic cancer treatment^3^,^10^,^11^. Although there is abundant evidence supporting the role of Stat3 in pancreatic cancer to justify the discovery of novel Stat3 therapeutics, only few show promising activity in terms of Stat3 inhibition *in vitro* and an associated antitumor effect^3^. LTP-1 was selected from a series of arylsulfonamide compounds, which was designed as an Stat3 targeting inhibitor previously^12^. In this study, we found that LTP-1 inhibited pancreatic cancer cell growth and caused mitotic arrest, by directly binding to tubulin and acted like a tubulin destabilizing agent. Furthermore, long-term treatment of LTP-1 may induce polyploidy followed by pancreatic cancer cell apoptosis. Significant antitumor activity of LTP-1 was also observed in a pancreatic xenograft model. Our data suggest that LTP-1 may be a promising therapeutic agent against pancreatic cancer.

## Results

### LTP-1 shows significant growth inhibition and causes cell cycle arrest in human pancreatic cancer cells

**Figure 1:**
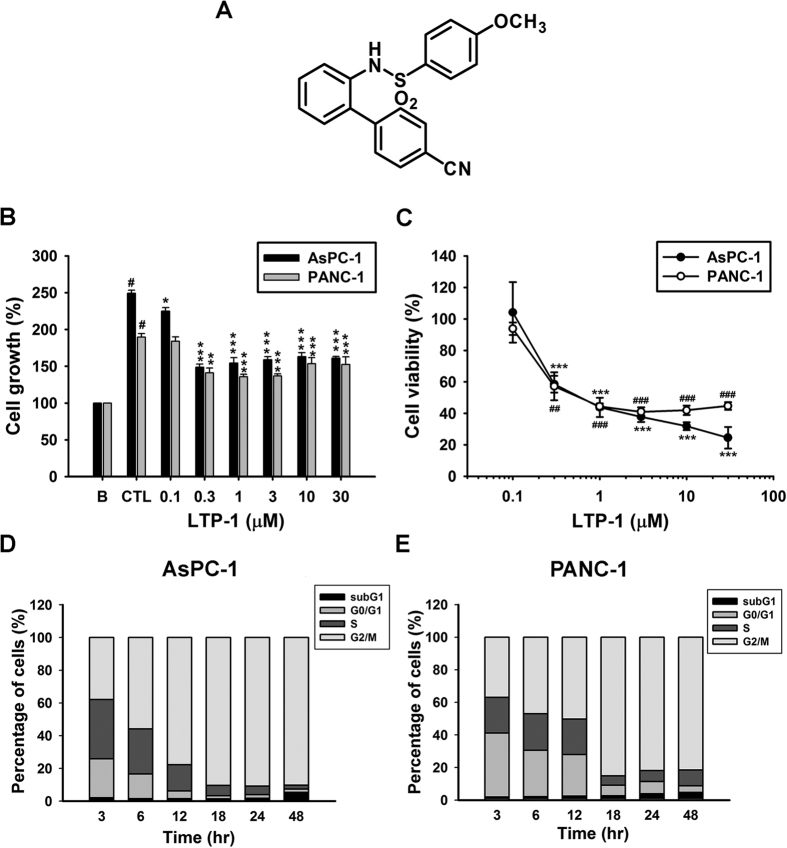
Effect of LTP-1 on cell growth, viability and cell cycle progression in pancreatic cancer cells. (**A**) Chemical structure of LTP-1. (**B**) Growth inhibition effect of LTP-1. Cells were incubated with or without LTP-1 at indicated concentration for 48 h, and then cell growth were measured by SRB assay. Data represent at least three independent experiments. ^#^*P* < 0.001 versus basal group; *P < 0.05; **P < 0.01; ***P < 0.001 versus control group. (**C**) Dose-dependent effect of LTP-1 on cell viability. Cells were incubated with or without LTP-1 at indicated concentration for 48 h, and then cell viability were measured by MTT assay. ** or ^##^*P* < 0.01; *** or ^###^*P* < 0.001 compared with control group. Data represent mean ± S.E.M. of at least three independent experiments. (**D,E**) Time-dependent cell cycle progression effect of LTP-1 in pancreatic cancer cells. Cells were treated with 1 μM LTP-1 for indicated time interval and cell cycle distribution was analyzed by flow cytometry. Similar results were obtained in at least three independent experiments.

Previously, we have screened growth inhibition activities of arylsulfonamide compounds designed as Stat3 inhibitors against pancreatic cancer cell lines^12^, as Stat3 activation is important for cancer progression^3^. According to the results of cell growth inhibition activities by using the SRB assay, LTP-1 showed most effective growth inhibition activity with GI_50_ values of 0.23 ± 0.01 and 0.42 ± 0.16 μM in AsPC-1 and PANC-1 cells, respectively (Fig. 1B). Furthermore, it showed significant concentration-dependent inhibition of cell viability with IC_50_ values of 0.76 ± 0.28 and 0.76 ± 0.23 μM in AsPC-1 and PANC-1 cells, respectively (Fig. 1C), whereas it showed no toxicity against normal epithelial cells (Supplementary Fig. S1). FACS cytometry was performed to analyze cell cycle progression and showed that LTP-1 substantially induced G_2_/M arrest in a time-dependent manner (Fig. 1D,E). Taken together, the findings suggest that LTP-1 showed profound growth inhibition and cytotoxicity with G_2_/M arrest in AsPC-1 and PANC-1 cells. Therefore, we choose LTP-1 to further evaluate its anticancer mechanisms in human pancreatic cancer cells.

### LTP-1 causes tubulin destabilization and activates G_2_/M cell cycle regulatory proteins

**Figure 2:**
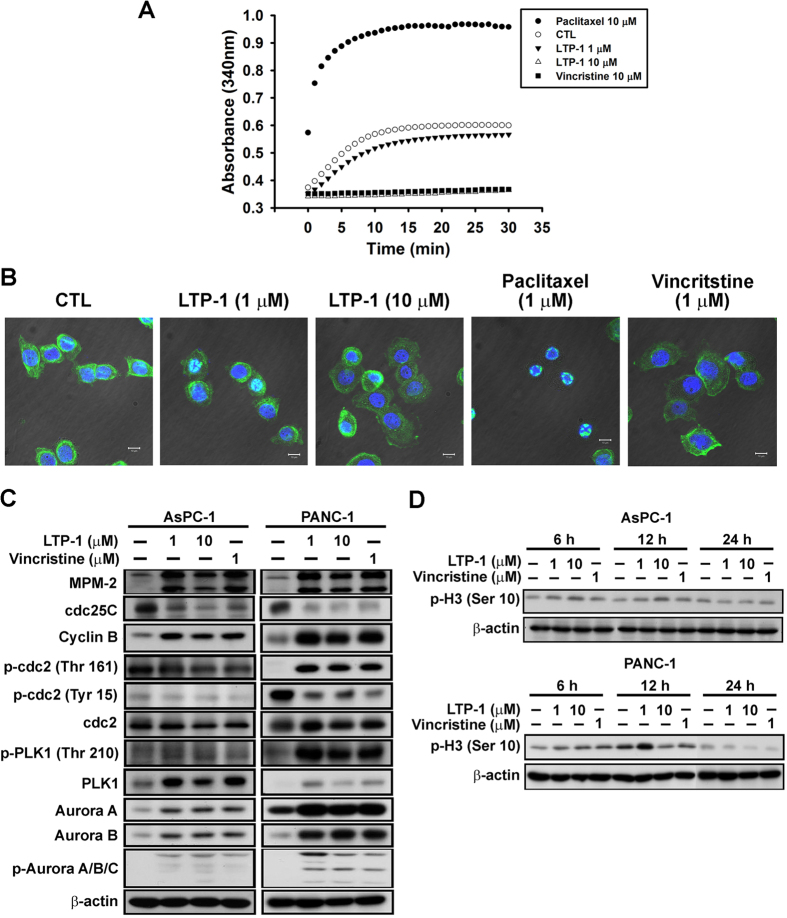
Effect of LTP-1 on tubulin destabilization and G2/M regulatory proteins. (**A**) Effect of LTP-1 on microtubule assembly *in vitro*. Purified tubulin was suspended in reaction buffer incubating at 37 °C with GTP in the absence or presence of LTP-1, paclitaxel or vincristine. Assembly of microtubules was determined by measuring absorbance at 340 nm using a spectrophotometer every 1 min for 30 min. (**B**) Effect of LTP-1 on tubulin distribution. AsPC-1 cells were treated with DMSO, LTP-1, paclitaxel or vincristine for 24 h. After treatment, microtubules were stained with β-tubulin and FITC-conjugated anti-mouse IgG (green fluorescence). Nuclear DNA was visualized by DAPI staining (blue fluorescence). Microtubule networks were analyzed with the Leica TCS SP2 Spectral Confocal System. Scale bar, 10 μm. (**C**) Effect of LTP-1 on G2/M cell cycle regulatory proteins. (**D**) Time-dependent effect of LTP-1 on Histone 3 phosphorylation. Both cells were treated with LTP-1 or vincristine for (**C**) 24 h or (**D**) indicated time interval, and then cell lysate were subjected to western blot analysis with indicated antibodies.

Typical tubulin binding agents such as paclitaxel and vincristine may induce mitotic arrest by affecting microtubule organization^5^. Thus, we performed an *in vitro* tubulin polymerization assay to examine whether LTP-1 may induce a similar effect. As shown in Fig. 2A, tubulin polymerization occurred under controlled conditions and LTP-1 acted like vincristine, to inhibit tubulin polymerization in a concentration-dependent manner. Similar results could be found using immunofluorescence *in situ* labeling of β-tubulin (Fig. 2B). Paclitaxel enhanced microtubule assembly and mitotic spindle formation, whereas LTP-1 and vincristine disrupted microtubule formation (Fig. 2A). These data indicated that LTP-1, which acted like vincristine, may promote tubulin destabilization and inhibit microtubule formation to cause mitotic arrest. We further examined the effect of LTP-1 on cell cycle regulatory proteins to characterize the mechanisms involved in LTP-1-induced G_2_/M arrest. As shown in Fig. 2C, LTP-1 increased the phosphorylation of MPM-2, which is a mitotic marker. Furthermore, LTP-1 inhibited both cdc25C expression and p-cdc2 (Tyr 15) phosphorylation, but increased cyclin B expression and p-cdc2 (Thr 161) phosphorylation, indicating that LTP-1 triggered mitotic arrest instead of G2 arrest. PLK, which is an M-phase-specific protein kinase, and Aurora kinases, which have been implicated in several vital events in mitosis, were also activated during LTP-1 treatment. LTP-1 also stimulated p-Histone 3 (Ser 10) phosphorylation, which is a G_2_/M transition marker, in a time-dependent manner (Fig. 2D). Taken together, these data suggest that LTP-1 may cause tubulin destabilization thereby inducing mitotic arrest in pancreatic cancer cell lines.

### LTP-1 inhibits Stat3 phosphorylation and is independent of the effect of LTP-1-induced mitotic arrest

**Figure 3:**
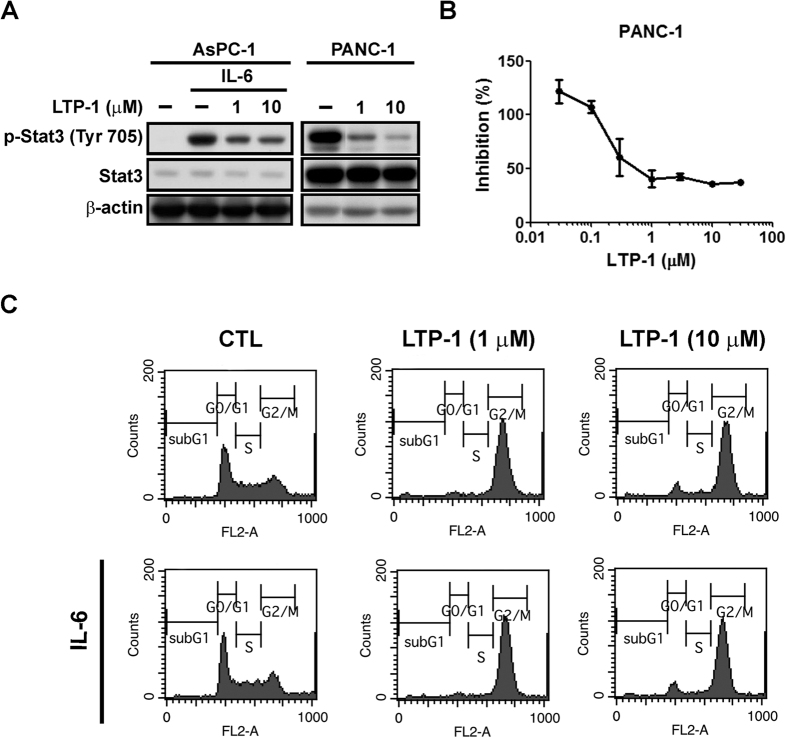
Effect of LTP-1 on Stat3 phosphorylation. (**A**) LTP-1 inhibits Stat3 phosphorylation. AsPC-1 cells were pretreated with IL-6 (10 ng/ml) for 30 min followed by LTP-1 treatment for 24 h whereas PANC-1 cells were treated with LTP-1 for 24 h. (**B**) Dose-dependent effect of LTP-1 on Stat3 activation. PANC-1 cells were treated with indicated concentrations of LTP-1 for 24 h. Whole cell lysate were subjected to p-Stat3 (Y705) detection using ELISA. (**C**) Correlation between Stat3 activation and LTP-1-induced G2/M arrest. AsPC-1 were stimulated with or without 10 ng/ml IL-6 for 30 min followed by treatment of LTP-1 for 24 h and cell cycle was analyzed by flow cytometry. Similar results were obtained in at least three independent experiments.

Since Stat3 activation plays an important role in pancreatic cancer progression, we further examined the ability of LTP-1 to inhibit Stat3 phosphorylation. AsPC-1, which is a Stat3 non-activated cell line, was stimulated with IL-6 to induce Stat3 phosphorylation (Supplementary Fig. S2). As shown in Fig. 3A, LTP-1 inhibited Stat3 phosphorylation in AsPC-1 and PANC-1 cells in a concentration-dependent manner. In PANC-1 cells, where Stat3 is highly activated, LTP-1 inhibited Stat3 phosphorylation in a concentration-dependent manner with an IC_50_ value of 0.61 ± 0.43 μM (Fig. 3B). Evidence suggests that Stat3 activation may contribute to cell cycle progression^13^; therefore, we wished to further determine whether LTP-1 caused mitotic arrest by deactivating Stat3. As shown in Fig. 3C, LTP-1-induced mitotic arrest was not affected by IL-6 stimulation in AsPC-1 cells, which indicates that LTP-1-induced mitotic arrest is independent of LTP-1-inhibited Stat3 phosphorylation. Namely, LTP-1 caused Stat3 deactivation, which played little role in LTP-1-induced mitotic arrest.

### ERK activation contributes little to LTP-1-induced mitotic arrest

**Figure 4:**
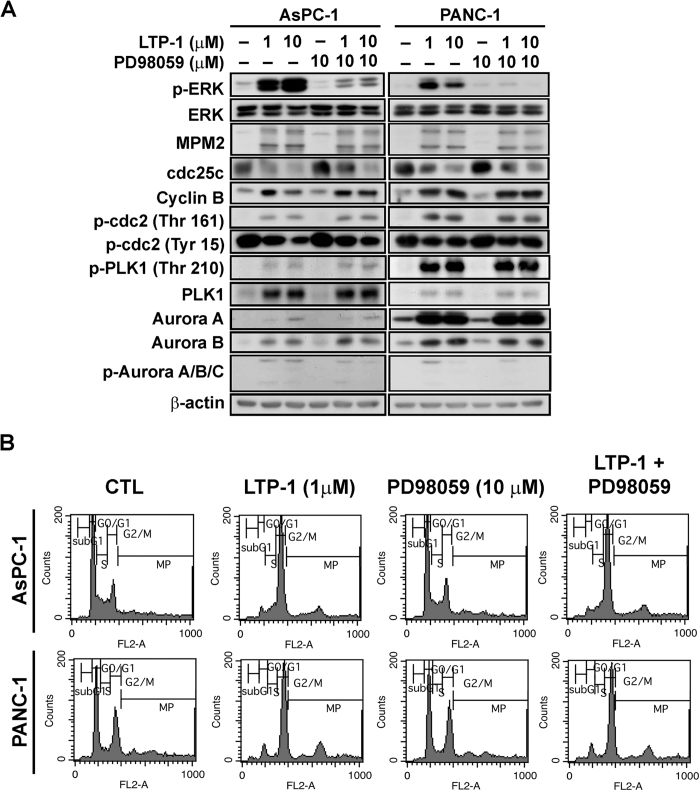
The role of ERK activation in LTP-1-induced mitotic arrest. Both cells were pretreated with 10 μM PD98059 for 2 h and then co-treated with various concentrations of LTP-1 for 24 h. (**A**) Whole cell lysates were subjected to western blot analysis. (**B**) Cell cycles were analyzed using FACS flow cytometry.

Evidence has shown that MAPK pathway activation causes G_2_/M arrest and affects mitotic spindle formation^14^,^15^,^16^. Thus, we further investigated whether LTP-1-induced mitotic arrest would be affected by ERK activation. As shown in Fig. 4A, LTP-1 indeed induced ERK activation. However, when we treated the cells with PD98059, an ERK inhibitor, to block LTP-1-induced ERK activation, LTP-1-activated expression of G_2_/M regulatory proteins could not be reversed. Further, PD98059 could not reverse LTP-1-induced mitotic arrest (Fig. 4B). The same phenomenon could be observed using ERK siRNA that ERK knockdown could not reverse LTP-1-induced G2/M arrest (Supplementary Fig. S3). Therefore, we concluded that ERK activation was not involved in LTP-1-induced mitotic arrest.

### Long-term treatment of LTP-1 induces multiploidy and apoptosis in human pancreatic cancer cells

**Figure 5:**
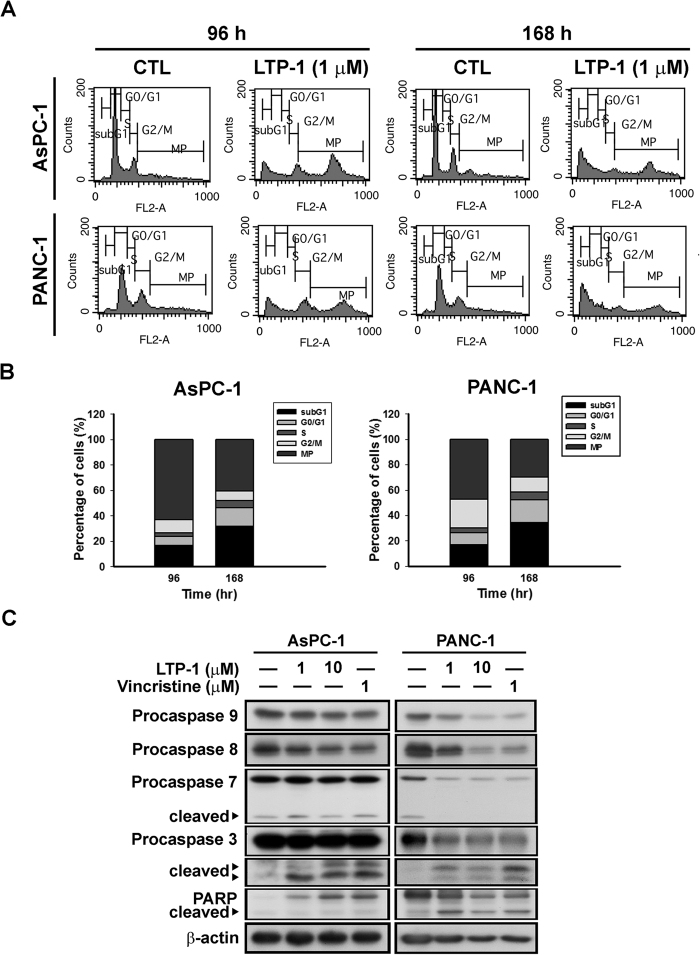
Long-term treatment of LTP-1 induced polyploidy and apoptosis in human pancreatic cancer cells. (**A**) LTP-1 induced polyploidy after long-term treatment of LTP-1. (**B**) Quantitative data of LTP-1-induced cell cycle distribution. Both cells were treated with LTP-1 for 96 or 168 h and cell cycles were analyzed by flow cytometry. (**C**) Long-term treatment of LTP-1 induced caspases and PARP activation. Both cells were treated with LTP-1 for 168 h and whole cell lysate were subjected to western blot analysis. Similar results were obtained in at least three independent experiments.

Since long-term exposure of tubulin-binding agents may induce multiploidy (MP)^13^, which promotes cell apoptosis in tumor cells^17^, we further examined the long-term effect of LTP-1 in pancreatic cancer cells. As shown in Fig. 5A,B, LTP-1 may induce multiploidy and increased subG1 cell population after long-term treatment, in both pancreatic cancer cell lines. Furthermore, long-term LTP-1 treatment could cause caspase and PARP activation and induce cell apoptosis (Fig. 5C). Thus, long-term treatment of LTP-1, a tubulin destabilizing agent, may induce MP in pancreatic cancer cells to eventually induce cell apoptosis.

### LTP-1 exhibits antitumor activity *in vivo*

**Figure 6:**
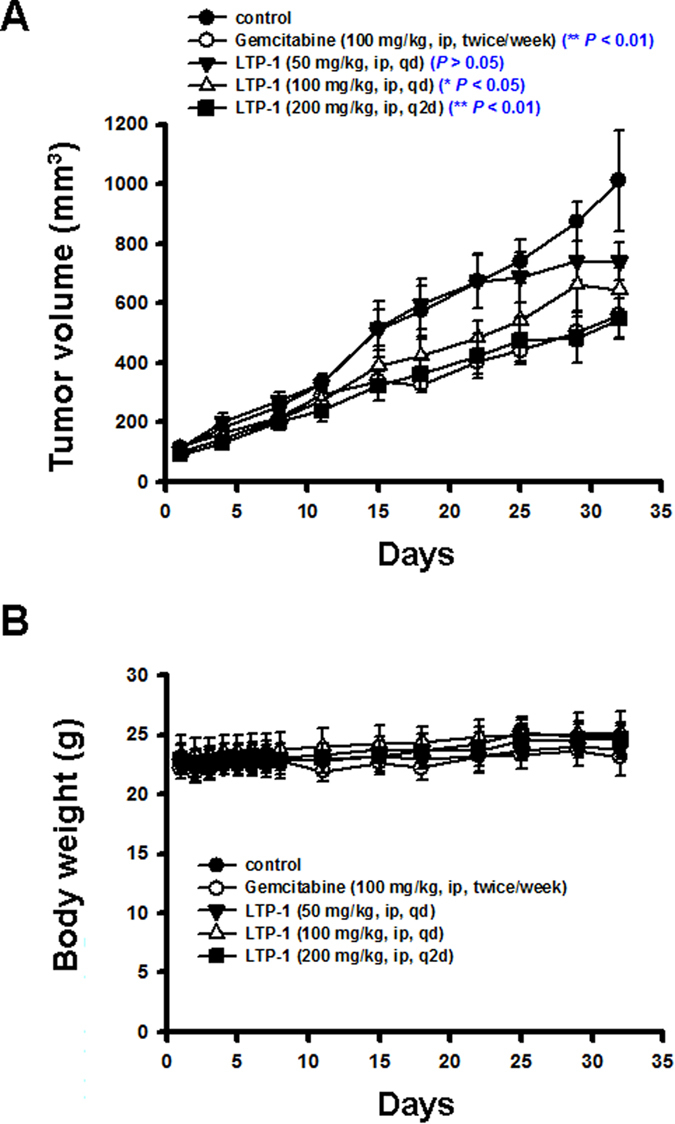
LTP-1 suppressed AsPC-1 human pancreatic adenocarcinoma growth *in vivo*. AsPC-1 xenograft nude mice were intraperitoneally (i.p.) administered LTP-1 (50, 100 or 200 mg/kg), Gemcitabine (100 mg/kg), or vehicle after tumor cell implantation. (**A**) LTP-1-inhibited tumor growth in mice. (**B**) body weight of tumor bearing mice under the treatment during the study. Data represent mean ± SEM from eight mice in each group. (*P* < 0.05 and *P* < 0.001 versus control group).

Since LTP-1 induced apoptosis *in vitro*, we further examined its antitumor effect in an AsPC-1 xenograft model *in vivo*. We intraperitoneally injected LTP-1 (50 mg/kg, qd; 100 mg/kg, qd and 200 mg/kg, q2d) in nude mice. Gemcitabine (100 mg/kg), the most common chemotherapy for pancreatic cancer, was used as a standard for comparison. As shown in Fig. 6A, tumor size was significantly inhibited in groups treated with higher doses of LTP-1, which showed a stronger inhibitory effect than that recorded in the gemcitabine-treated group. Both these treatments did not affect body weight of the animals (Fig. 6B), indicating that LTP-1 exhibited little apparent toxicity *in vivo*.

## Discussion

Pancreatic cancer remains one of the most lethal types of cancer, whose mortality rates are increasing despite significant advancement in multiple therapeutic strategies. Developing new anticancer agents against pancreatic cancer is therefore an important issue. Here, we reported that LTP-1, a novel arylsulfonamide, exhibited tubulin binding ability to induce mitotic arrest in pancreatic cancer cell, and polyploidy followed by apoptosis after long-term treatment. Furthermore, LTP-1, which was first designed as a Stat 3 inhibitor^12^, inhibited Stat 3 phosphorylation and inhibited tumor growth significantly in a pancreatic xenograft model *in vivo*. These results suggest that LTP-1 could be a therapeutic option for pancreatic cancer treatment.

Constitutive activation of Stat3 is implicated in a wide range of human cancers, which indicates that Stat3 is a target for cancer treatment. Besides its transcriptional activity, Stat3 has also been reported to physically associate with tubulin to regulate microtubule function and Stat3 activaiton would antagonize with stathmin to cause tubulin polymerization^18^,^19^; some observations provide insight into potential mechanisms by which microtubule-targeting agents exert their Stat3 inhibitory effects^20^,^21^, which also reduce the expression of Stat3 target genes and correlate with its cytotoxic effect^22^. According to our data, LTP-1, which was first designed as a Stat3 inhibitor, directly interacted with and destabilized tubulin (Fig. 2A), and also inhibited Stat3 phosphorylation *in vitro* (Fig. 3A,B). However, even though we induced Stat3 activation by IL-6 treatment in non-Stat3 activated AsPC-1 cells, the LTP-1-induced mitotic arrest won’t be affected (Fig. 3C), which indicated the parallel of LTP-1-induced mitotic arrest and LTP-1-inhibited Stat3 phosphorylation. In other words, LTP-1 might exhibit dual inhibitory function on both tubulin and Stat3. Nevertheless, the cell population of G2/M was significantly induced under 1 μM and 10 μM LTP-1 treatments at nearly the same level (Fig. 3C and Supplementary Fig. S3), which coincided with that two concentrations of LTP-1 showed no concentration-dependent effect in the changes of cdc25C, p-cdc2 (Tyr15), p-cdc2 (Thr161) and cyclin B (Fig. 2C). In contrast, dose-dependent effect of tubulin depolymerization could be seen in Fig. 2A using a cell-free tubulin polymerization assay. Since Stat3 and tubulin have been reported that physically associate with each other^18^,^19^, LTP-1, which was previously designed as a Stat3 inhibitor^12^, may cause tubulin depolymerization partly due to Stat3 inhibition effect in cell level and lead to potentiate the LTP-1-induced mitotic arrest to reach plateau phase that no dose-dependent effect could be seen under LTP-1 treatment (Fig. 3C). The interaction between Stat3 and tubulin may also lead to difficulties to distinguish the dual effect of these compounds; therefore, detailed mechanisms of the relationship between tubulin and Stat3 under LTP-1 treatment should be further elucidated.

Evidence has shown that paclitaxel causes G_2_/M cell cycle arrest in HeLa cells through ERK activation^14^ and activation of ERK in response to microtubule binding agents has been reported^23^,^24^,^25^. Therefore, we wanted to further determine whether LTP-1-induced ERK activation is the main reason that leads to cell cycle arrest in LTP-1-treated pancreatic cancer cells. According to our data, LTP-1 indeed induced ERK activation; however, the ERK inhibitor PD98059 and siRNA ERK could not reverse LTP-1-induced G_2_/M arrest and G_2_/M associated proteins expression (Fig. 4A,B and Supplementary Fig. S3). β-Sitosterol has been reported to cause cell cycle arrest in the G_2_/M phase, endoreduplication, and eventually apoptosis. Although β-sitosterol induces ERK activation, the significance of ERK activation in β-sitosterol-induced apoptosis and endoreduplication is unknown^26^. Previous evidence has shown that tubulin depolymerization agents such as nocodazole^27^ and DYZ-2-90^28^ would induce sustained ERK activation until 24–36 hours after treatment; and ERK has been reported to be responsible for cell survival in order to protect cells from microtubule-interfering agents-induced cell death during mitotic stress^29^,^30^. Although mitotic arrest triggered by LTP-1 is independent of ERK activation, according to these literatures, we may predict that LTP-1 might also exert its sustained ERK activation as a surviving signal to overcome LTP-1-induced mitotic stress. However, the exact role of ERK on LTP-1-induced cell apoptosis in pancreatic cancer still needs to be further investigated.

Polyploidy, which accords with haploid number of chromosomes, could be induced by mitotic spindle function failure, known as mitotic slippage, leading to accumulation of tetraploid cells^31^. Evidence has shown that p53-dependent G_1_ post-mitotic checkpoint would cause tetraploid cell arrest at the G_1_ phase to prevent entry into the S phase, leading to formation of polyploidy^32^,^33^. However, dysfunction of the checkpoint would cause tetraploid cells to maintain their proliferative potential and polyploidization. Pancreatic cancer cells used in this study harbor mutant p53, which may explain why polyploidy occurs in these cells after LTP-1 long-term treatment. According to our data, p53 mutant pancreatic cancer cells show formation of polyploidy after LTP-1 treatment for 96 h, and show increased induction of subG_1_ cell population, caspase activation, and eventually apoptosis after treatment for 168 h.

Gemcitabine is the most frequently used chemotherapy, alone or in combination treatment, for pancreatic cancer. Tubulin binding agents such as albumin-bound formulations of paclitaxel have also been approved by the FDA for pancreatic cancer treatment. However, the clinical outcome, overall survival rate, and development of in pancreatic cancer still remain disappointing factors^34^. Therefore, developing an ideal therapeutic agent against pancreatic cancer is an urgent medical need. According to our data, LTP-1 showed a significant tumor suppressive effect in the AsPC-1 xenograft model with no obvious toxicity, which indicated its possible role in pancreatic cancer treatment. Furthermore, the combination of LTP-1 with standard therapies such as gemcitabine may also be a therapeutic option for overcoming resistance in pancreatic cancer. However, detailed mechanisms and function should be further investigated.

## Conclusion

The present study indicates that LTP-1, a novel synthetic arylsulfonamide derivative, acts by suppressing pancreatic cancer cell growth, inducing tubulin destabilization, and may exhibit a dual effect to inhibit Stat3 phosphorylation. It may also cause polyploidy, caspase activation, and eventually apoptosis in pancreatic cancer cells. *In vivo* data suggest that LTP-1 reduced tumor volume in a pancreatic cancer xenograft model without significant toxicity. These results suggest that LTP-1 might be a possible therapeutic option and worthy of further development as an anticancer agent for pancreatic cancer treatment.

## Materials and methods

### Cell culture and reagents

Two human pancreatic cancer cell lines, AsPC-1 (Stat3 non-active) and PANC-1 (Stat3 constitutive active), were purchased from American Type Culture Collection (Manassas, VA, USA). Human colon epithelial cells FHC were a kind gift from Dr. Ya-Wen Cheng (Taipei Medical University, Taipei, Taiwan). AsPC-1 cells were cultured in RPMI-1640, PANC-1 cells were cultured in DMEM and FHC cells were cultured in DMEM/F12 (supplement with 25 mM, 10 ng/ml cholera toxin, 0.10 μg/ml insulin, 0.005 mg/ml transferrin, 0.5 μg/ml hydrocortisone, 20 ng/ml EGF, 1% sodium pyruvate and 1% NEAA). All medium contained 10% fetal bovine serum and penicillin (100 units/ml)/streptomycin (100 μg/ml)/amphotericin B (0.25 μg/ml). All cells were maintained in humidified air containing 5% CO_2_ at 37 °C. Cell density was maintained between 1 × 10^5^ and 1 × 10^6^ cells/ml and passaged every 2 to 3 days.

LTP-1 and LTP-1 derivatives were newly synthesized arylsulfonamide compounds provided by Prof. Jing-Ping Liou (Taipei Medical University, Taipei, Taiwan). Propidium iodide, 3-(4,5-Dimethylthiazol-2-yl)-2,5-diphenyl-tetrazolium bromide (MTT), sulforhodamine B (SRB), PD98059, and all of the other chemical reagents were obtained from Sigma (St Louis, MO, USA). Dulbecco’s Modified Eagle Medium (DMEM), RPMI-1640 medium, fetal bovine serum (FBS), trypsin-EDTA, sodium pyruvate solution 100 mM were purchased from GIBCO/BRL Life Technologies (Grand Island, NY, USA). Penicillin/streptomycin/amphotericin B solution was obtained from Biological Industries Ltd. (Kibbutz Beit-Haemek, Isarel) IL-6 was purchased from Millipore (Temecula, CA, USA). The following antibodies were used: p-cdc2 (T161), p-cdc2 (Y15), Aurora A, Aurora B, p-Aurora A (Thr288)/B (Thr232)/C (Thr198), PLK1, p-Erk1/2 (Thr202/Tyr204), Erk, caspase-8, and caspase-9 (Cell Signaling Technology, Beverly, MA, USA); cyclin B, cdc25C, cdc2, and PARP (Santa Cruz Biotechnology, Santa Cruz, CA, USA); p-MPM-2 (Ser/Thr) and p-Histone 3 (S10) (Upstate Biotechnology Inc., Lake Placid, NY, USA); p-PLK (T210), STAT3, and caspase 7 (BD Bioscience, San Hose, CA, USA); β-actin (Millipore, Temecula, CA, USA); caspase-3 (Imgenex, San Diego, CA, USA); anti-β-tubulin, FITC-conjugated anti-mouse IgG (Sigma Chemical, St Louis, MO, USA). 4′,6-diamidino-2-phenylindole (DAPI) was obtained from Roche Diagnostics Corporation (Indianapolis, IN, USA).

### SRB assay

AsPC-1 and PANC-1 cells were seeded in 96-well plates overnight. Cells were fixed with 10% trichloroacetic representing cell population at the time of drug treatment. After incubation with vehicle or LTP-1 for 48 h, cells were fixed with 10% trichloroacetic acid and then stained with SRB at 0.4% (w/v) in 1% acetic acid. Excess SRB was washed away by 1% acetic acid and cells were lysed with 10 mM Trizma base. The absorbance was measured at wavelength of 515 nm. Growth inhibition of 50% (GI_50_) is determined at the drug concentration that results in 50% reduction of total protein increase in control cells during the compound incubation.

### MTT assay

Cell viability was assessed by mitochondria dehydrogenases activity, forming an insoluble blue formazan product after reducing tetrazolium ring of MTT. AsPC1, PANC-1 and FHC cells were plated in 96-well plate (5000 cells/well) and treated with 200 μl of different doses of vehicle or LTP-1 for 48 h. After treatment, 100 μl of 0.5 mg/ml MTT were added to each well and incubated at 37 °C for 1 h. MTT-containing medium were removed and 100 μl DMSO were added to each well to lyse cells. Plates were then measured at 550 nm using an enzyme-linked immunosorbent assay (ELISA) reader (Packard, Meriden, CT, USA).

### Flow cytometry analysis

Cells were treated with indicated condition and then harvest and suspended using 75% ethanol and store at 4 °C overnight. After centrifugation, fixed cells were incubated with DNA extraction buffer (0.2 M Na_2_HPO_4_, 0.1 M citric acid, pH 7.8) at room temperature for 30 min. Cells were centrifuged and resuspended in PI staining buffer (100 μg/ml RNase, 80 μg/ml PI and 0.1% Triton X-100 in darkness). The DNA content was analyzed using a FACScan flow cytometer with CellQuest software (Becton Dickinson, San Jose, CA, USA).

### Tubulin polymerization assay

Microtubule assembly was assessed using the CytoDYNAMIX Screen kit (BK006P; Cytoskeleton Inc., Denver, CO, USA). Purified porcine tubulin proteins (>99% purity) were suspended in G-PEM buffer containing 80 mM PIPES, 2 mM MgCl_2_, 0.5 mM EGTA, 1 mM GTP (pH 6.9) and 15% glycerol in the absence or presence of indicated compounds at 4 °C. The mixture was immediately transferred to pre-warmed 96-well plates, and absorbance was measured at 340 nm every 1 min for 30 min using a 37 °C plate reader (SpectraMAX Plus; Molecular Devices Inc., Sunnyvale, CA, USA).

### Immunofluorescence

Cells were seeded on chamber slide and treated with indicated condition. Cells were washed with PBS for 3 times and fixed with −20 °C methanol at 4 °C for 10 min and then blocked with 2% BSA in PBS at 37 °C for 1 h. After PBS washing, cells were incubated with indicated primary antibody at 4 °C overnight. After PBS washing, secondary antibody FITC-conjugated DAPI containing IgG were added and incubated for 45 min at 4 °C in dark. Slides were imaged using a Leica TCS SP2 confocal spectral microscope (Buffalo, NY, USA).

### Protein extraction and western blot

Cells were treated with indicated condition and then harvest in lysis buffer (5 mM NaF, 1 mM Na_3_VO_4_, 10 μg/ml leupeptin, 1 mM PMSF, 10 μg/ml aprotinin, 20 mM Tris-HCl buffer pH7.5, 150 mM NaCl, 1 mM EDTA, 0.5 mM EGTA, 1% Nonidet P-40 and 0.5% Na deoxycholate) incubating on ice for 30 min followed by centrifugation at 13000 rpm for 30 min. Total protein was determined and equal amounts of protein were separated by 8–15% sodium dodecyl sulfate-polyacrylamide gel electrophoresis (SDS–PAGE) and transferred to poly(vinylidene difluoride) (PVDF) membranes. Membranes were immunoblotted with specific antibodies overnight at 4 °C and then applied to appropriate horseradish peroxidase-conjugated anti-mouse or anti-rabbit IgG secondary antibodies for 1 h at room temperature. Signals were visualized using an enhanced chemiluminescence (Amersham, Buckinghamshire, UK).

### STAT3 ELISA assay

PANC-1 cells were treated with different concentrations of LTP-1 and for 24 h. Cells were harvested and equivalent aliquots of protein were subjected to a PathScan^®^ phospho-Stat3 (Tyr705) sandwich ELISA kit (Cell Signaling Technology) following manufacture’s instruction. In briefly, whole cell lysate was first half-diluted with sample diluent and incubated overnight at 4 °C. Wells were washed with wash buffer and p-Stat3 detection antibody was added. After incubating 1 h at 37 °C, antimouse IgG HRP-linked antibody was added incubating 30 min at 37 °C. After incubation, TMB substrate was added for color production and STOP solution was added for reaction stop. Results were measured by spectrophotometer at wavelength 450 nm.

### Transient transfection

Silencer select siRNA against ERK was purchased from Ambion (Austin, TX, USA). AsPC-1 and PANC-1 cells were transfected with Lipofectamine 2000 transfection reagent (Invitrogen) according to the manufacturer’s instruction. Transfected cells were grown for 24 h, treated with LTP-1 for another 24 h and then harvested for western blot analysis.

### *In vivo* xenograft model

Female nude mice were injected subcutaneously with the same volume of BD Matrigel Matrix HC (catalog 354248, BD bioscience), and AsPC1 cells (1 × 10^7^ cell/mouse) into the flank of each animal. When the tumors had grown to around 100 mm^3^, animals were divided into five groups (n = 8) and receive the following treatment by intraperitoneal injection for 32 days during the study: (a) vehicle alone, (b) Gemcitabine at 100 mg/kg twice a week, (c) LPT-1 at 50 mg/kg daily, (d) LTP-1 at 100 mg/kg daily and (e) LTP-1 200 mg/kg, every other day. LTP-1 was dissolved in vehicle (0.5% cremophor EL + 0.5% DMSO + 90% dextrose). Tumor size was measured twice weekly and calculated from V = l*w^2^/2, where w = width (w) and l = length (l). All animal experiments were performed in accordance with guidelines and regulations followed ethical standards, and protocols has been reviewed and approved by Animal Use and Management Committee of Taipei Medical University (LAC-2013-0139).

### Statistical analysis

Each experiment was performed independently at least three times and the data are presented as mean ± SEM for the indicated number of separate experiments. Student’s t-test was used to compare the mean of each group with that of the control group in experiments and one-way ANOVA was used in animal study. P-values less than 0.05 were considered significant (*P < 0.05, **P < 0.01, ***P < 0.001).

## Additional Information

**How to cite this article**: Huang, H.-L. *et al*. LTP-1, a novel antimitotic agent and Stat3 inhibitor, inhibits human pancreatic carcinomas *in vitro* and *in vivo. Sci. Rep.* **6**, 27794; doi: 10.1038/srep27794 (2016).

## Supplementary Material

Supplementary Information
