# Supplementary material for: LTP-1, a novel antimitotic agent and Stat3 inhibitor, inhibits human pancreatic carcinomas in vitro and in vivo
Source: Sci Rep. 2016 Jun 9;6:27794. doi: 10.1038/srep27794 (PMC4899784; doi:10.1038/srep27794)
Supplement: Supplementary Information [file srep27794-s1.doc]

**LTP-1, a novel antimitotic agent and Stat3 inhibitor, inhibits human pancreatic carcinomas *in vitro* and *in vivo***

Han-Li Huang^1,#^, Min-Wu Chao^1,#^, Chung-Chun Chen^2^, Chun-Chun Cheng^1^, Mei-Chuan Chen^3,4^, Chao-Feng Lin^1,5^, Jing-Ping Liou^6^, Che-Ming Teng^2^ and Shiow-Lin Pan^1,7*^

*^1^ The Ph.D. Program for Cancer Biology and Drug Discovery, College of Medical Science and Technology, Taipei Medical University, Taipei, Taiwan ^2^Pharmacological Institute, College of Medicine, National Taiwan University, Taipei, Taiwan*

*^3^Ph.D. Program for the Clinical Drug Discovery from Botanical Herbs, College of Pharmacy, Taipei Medical University, Taipei, Taiwan*

*^4^ Graduate Institute of Pharmacognosy, College of Pharmacy, Taipei Medical University, Taipei, Taiwan*

*^5^ Department of Internal Medicine, Division of Cardiology, Shuang Ho Hospital, Taipei Medical University, New Taipei City, Taiwan*

*^6^School of Pharmacy, College of Pharmacy, Taipei Medical University, Taipei, Taiwan*

*^7^Department of Pharmacology, College of Medicine, Taipei Medical University, Taipei, Taiwan*

^#^ These authors contributed equally to this work.

***Correspondence:**

**Shiow-Lin Pan, Ph.D.**

The Ph.D. Program for Cancer Biology and Drug Discovery,

College of Medical Science and Technology, Taipei Medical University,

No. 250 Wuxing Street, Taipei 11031, Taiwan

E-mail: [slpan@tmu.edu.tw](mailto:slpan@tmu.edu.tw)

Phone: 886-2-27361661 ext 7671


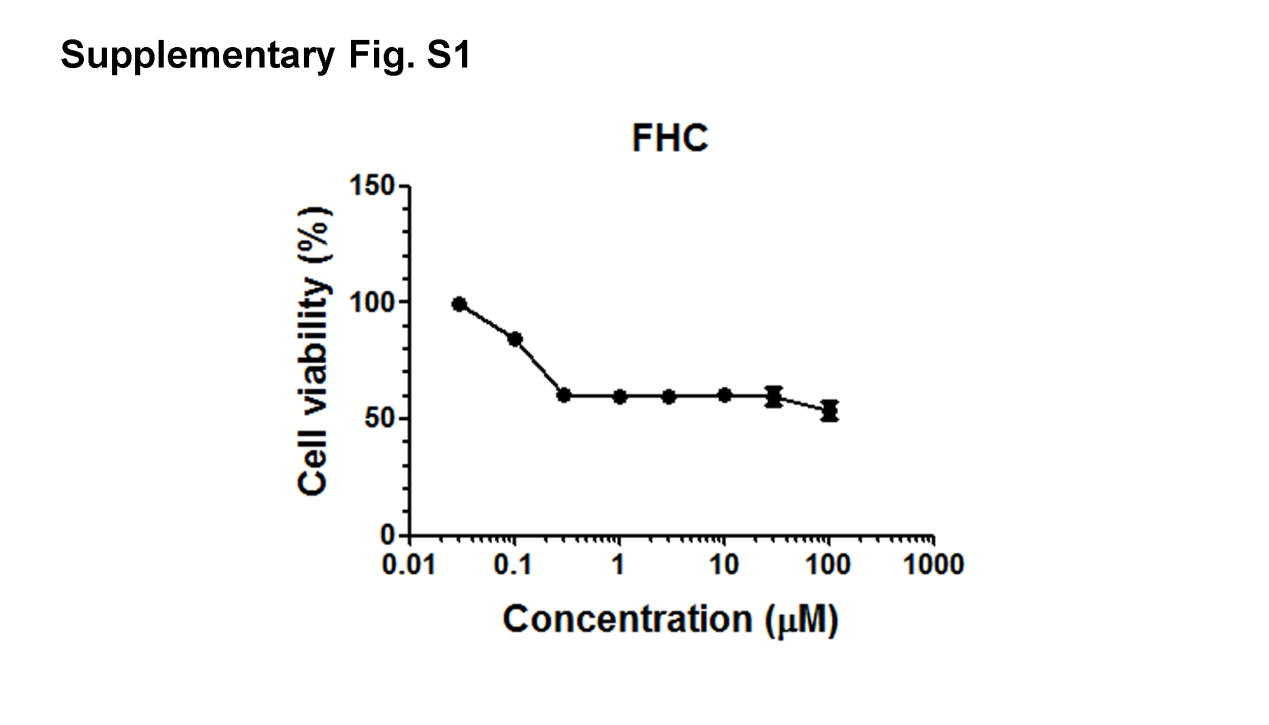
**Supplementary Fig. S1. The effects of LTP-1 on cell viability in human FHC cells.** Cells were incubated without or with the indicated concentrations of LTP-1 for 48 h and cell viability was evaluated by MTT assay. Data were expressed as mean ± S.E.M. of at least 3 independent experiments.

**
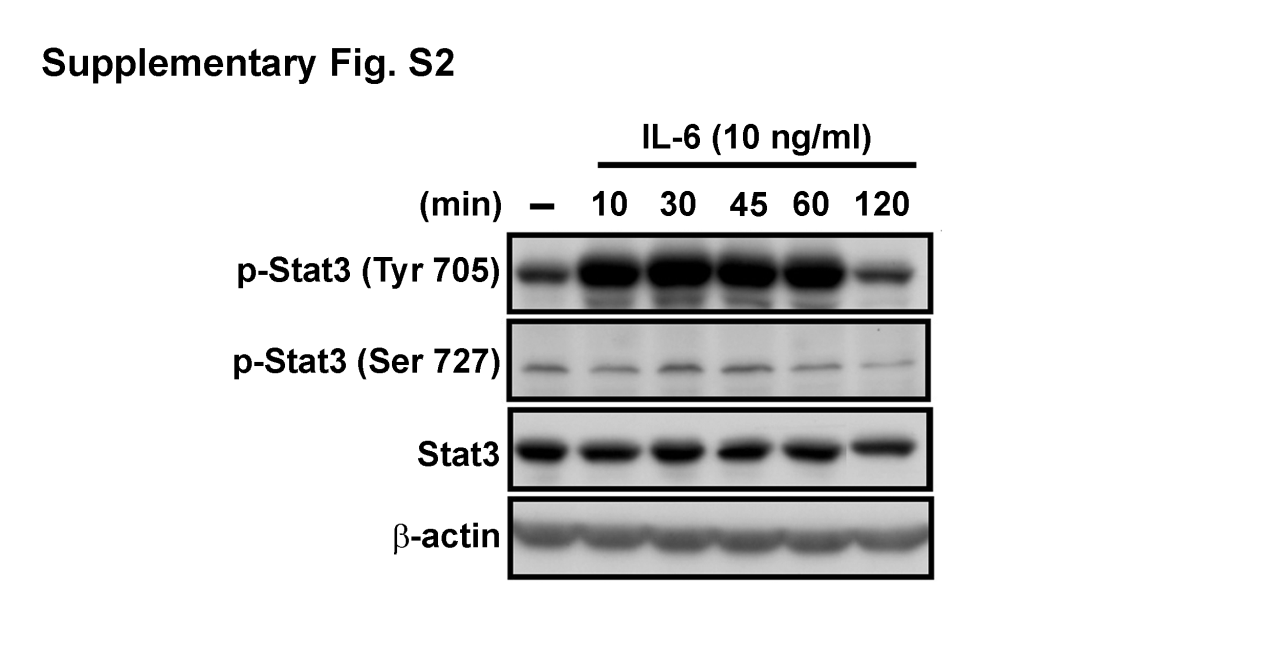
**

**Supplementary Fig. S2. Time-dependent effect of IL6 induced Stat3 activation in AsPC-1 cells.** AsPC-1 cells were treated with 10 ng/ml IL-6 for indicated time interval. Whole cell lysates were then subjected to western analysis. Similar results were obtained in at least three independent experiments.


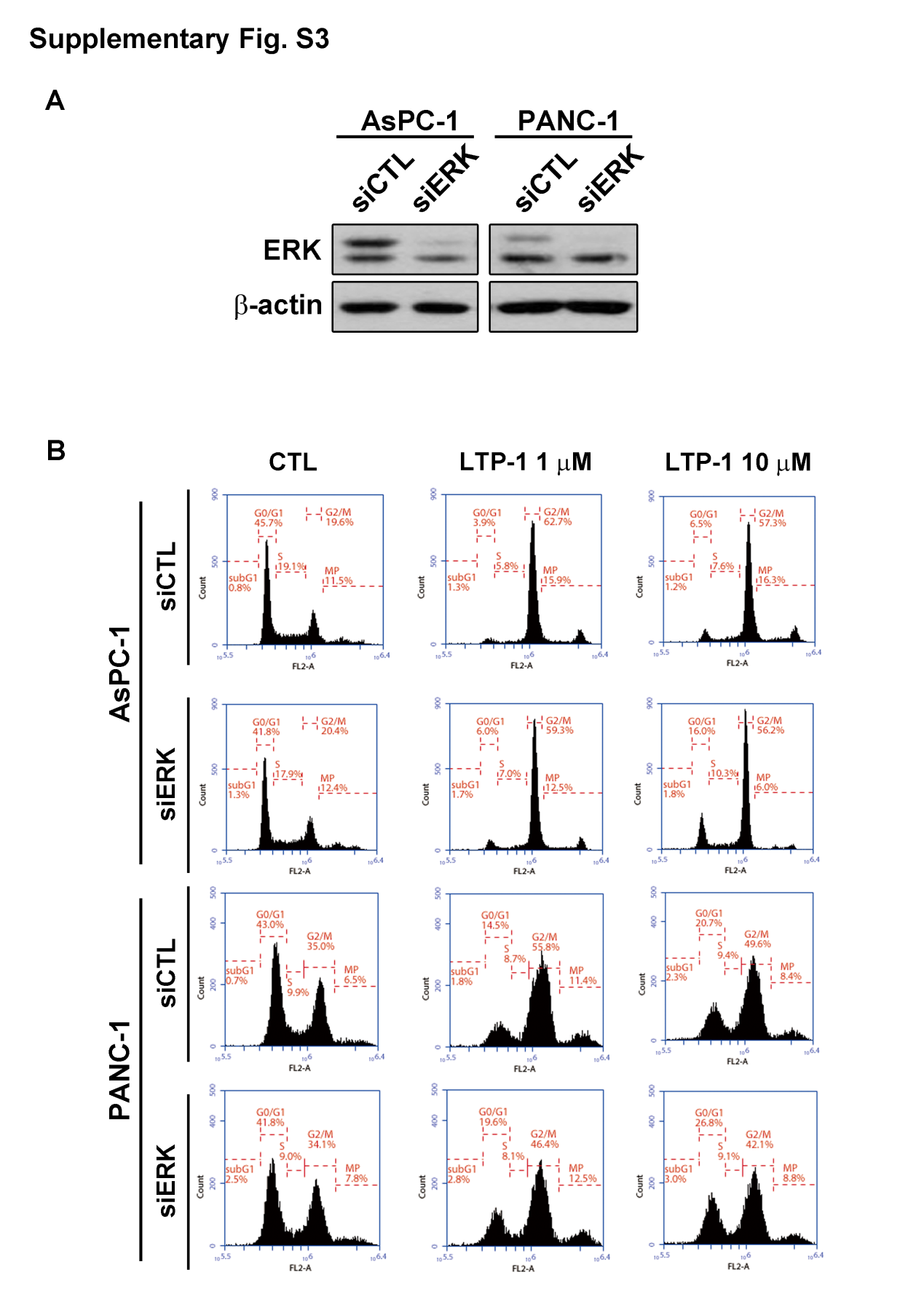
**Supplementary Fig. S3. The role of ERK in LTP-1-induced mitotic arrest.** Cells were transfected with control siRNA (siCTL) or ERK siRNA (siERK) and then treated with LTP-1 for 24 h. (A) Transfection efficiency was confirmed by western blot analysis. (B) LTP-1-induced cell cycle progression was analyzed using flow cytometry. Results are obtained from at least three independent experiments.
